# A genome-wide screen of bacterial mutants that enhance dauer formation in *C. elegans*

**DOI:** 10.1038/srep38764

**Published:** 2016-12-13

**Authors:** Amit Khanna, Jitendra Kumar, Misha A. Vargas, LaKisha Barrett, Subhash Katewa, Patrick Li, Tom McCloskey, Amit Sharma, Nicole Naudé, Christopher Nelson, Rachel Brem, David W. Killilea, Sean D. Mooney, Matthew Gill, Pankaj Kapahi

**Affiliations:** 1 Buck Institute for Research on Aging, 8001 Redwood Blvd, Novato, USA; 2 Nutrition & Metabolism Center, Children’s Hospital Oakland Research Institute, 5700 Martin Luther King Jr. Way, Oakland, CA, USA; 3 Department of Biomedical Informatics and Medical Education, University of Washington, Seattle, Washington 98195, USA; 4 Department of Metabolism & Aging, The Scripps Research Institute- Scripps Florida, Jupiter, Florida, 33458, USA

## Abstract

Molecular pathways involved in dauer formation, an alternate larval stage that allows *Caenorhabditis elegans* to survive adverse environmental conditions during development, also modulate longevity and metabolism. The decision to proceed with reproductive development or undergo diapause depends on food abundance, population density, and temperature. In recent years, the chemical identities of pheromone signals that modulate dauer entry have been characterized. However, signals derived from bacteria, the major source of nutrients for *C. elegans*, remain poorly characterized. To systematically identify bacterial components that influence dauer formation and aging in *C. elegans*, we utilized the individual gene deletion mutants in *E. coli* (K12). We identified 56 diverse *E. coli* deletion mutants that enhance dauer formation in an insulin-like receptor mutant (*daf-2*) background. We describe the mechanism of action of a bacterial mutant *cyaA*, that is defective in the production of cyclic AMP, which extends lifespan and enhances dauer formation through the modulation of TGF-β (*daf-7*) signaling in *C. elegans.* Our results demonstrate the importance of bacterial components in influencing developmental decisions and lifespan in *C. elegans*. Furthermore, we demonstrate that *C. elegans* is a useful model to study bacterial-host interactions.

The soil-dwelling nematode, *C. elegans*, assesses environmental cues including food availability, temperature, and population density^1^,^2^, to determine the choice between reproduction and survival^2^. Under favorable conditions, *C. elegans* attains reproductive maturity, but under adverse environments, worms enter dauer diapause, a long-lived stress resistant stage^2^. There are at least two types of chemical signals that dictate this developmental decision; a) dauer pheromones, a complex mixture of small molecules called ascarosides whose levels depend on worm population density^3^,^4^,^5^,^6^, and b) signals derived from bacteria that promote development^3^.

To detect microbial metabolites in the environment *C. elegans* has evolved multiple mechanisms, which allow discrimination between bacterial species that are appropriate food sources and those that are pathogenic or inferior in quality^7^,^8^. The chemosensory system senses bacterial metabolites in the local environment and modulates the organismal response to signaling through conserved molecular pathways^1^,^2^,^9^. These include the transforming growth factor beta (TGF-β), guanylyl cyclase, and insulin-like signaling (ILS) pathways, which ultimately converge to modulate the activity of key factors such as forkhead transcription factor (DAF-16/FOXO)^2^,^3^,^4^,^10^,^11^,^12^. The activity of DAF-16 is pivotal in determining the organism’s response, to either engage in reproductive growth or to enter dauer arrest state^2^,^13^. Although the genetic pathways that modulate dauer formation are relatively well understood, the identities of the signals that influence dauer formation remain poorly defined. *C. elegans* is known to respond to signals emanating from bacteria to detect food sources^8^, and a recent report identified bacterial fatty acids as a signal that modulates recovery from the dauer stage^14^. It is likely that the worm integrates a multitude of bacterial signals through the chemosensory system to make appropriate developmental decisions which remain undiscovered.

Dauer formation provides an easy to assess, binary readout of the type of food signals emanating from bacteria. Here we demonstrate how multiple individual gene knockouts in *E. coli* can influence development and survival in *C. elegans*. Through a systematic screen of ~4000 single gene knockout *E. coli* strains, we identified 56 *E. coli* mutants that enhanced dauer formation in a *C. elegans* insulin-like receptor mutant, *daf-2*, some of which also extended adult lifespan in control worms. One of the largest lifespan extensions was observed by feeding the bacterial mutant, *cyaA* (adenylate cyclase). We identify that *cyaA* influences TGF-β signaling to modulate dauer formation and lifespan. Our results demonstrate that the combination of bacterial and worm genetics can be a powerful tool to study bacterial-host interactions that influence nutrient signaling pathways and organismal physiology that may be relevant for host-microbiome interactions in other species.

## Results

### Bacterial mutants enhance dauer formation in *daf-2 (e1370*) mutants

To systematically study bacterial products that could act as food signals in *C. elegans*, we fed worms with bacterial strains from the *E. coli* Keio knockout library. The library consists of 3985 single-gene in-frame knockout mutants, with individual genes precisely excised and replaced with a Kanamycin resistance cassette in a K-12 BW25113 background^15^. To study the impact of bacteria on organismal physiology we screened for bacterial mutants that enhance dauer formation in a *daf-2(e1370*) mutant background. This worm strain carries a hypomorphic mutation in the insulin receptor ortholog gene that results in reduced flux through the ILS pathway and triggers constitutive dauer formation at high temperatures but undergoes normal development at low temperatures^12^,^16^,^17^,^18^,^19^. We carried out the screen at a semi-permissive temperature (21.5 °C), which allowed most worms on the control K-12 *E. coli* strain to develop to adulthood, with a small percentage entering the dauer stage. This assay created a sensitive readout to detect bacterial food signals in the diet, which influence organismal physiology detected by an increase in dauer formation in the worm.

**Figure 1:**
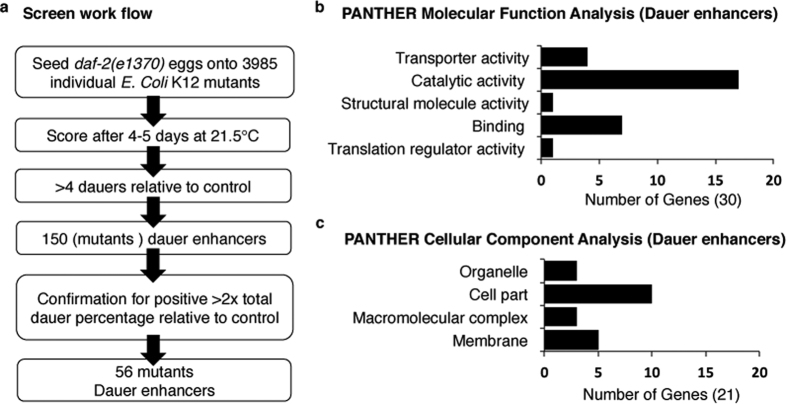
Schematic of the *E. coli* knockout screen for dauer formation in *C. elegans* and functional classifications of identified bacterial genes. (**a**) Schematic of the workflow of *E. coli* knockout screen for dauer enhancement in *C. elegans.* The screen resulted in 56 mutant bacterial strains enhancing dauer formation in the worm. The knockout candidates and associated statistical information are available on Table 1. (**b**) Functional classification of candidate genes (dauer enhancers) from the screen. The molecular function analysis of the candidate genes was done using PANTHER pathway database. (**c**) The cellular component analysis of these genes was done using PANTHER pathway database. For details pertaining to biological function analysis and enrichment analysis see Fig. S1.

In the primary screen, approximately 60 eggs were seeded onto plates with mutant *E. coli* strains and monitored for dauer formation after 4–5 days. The total number of dauers on the K-12 control strain was used as a reference and knockout strains that produced at least four more dauer animals than the control strain were marked as positive candidates and retested. This second, and more stringent, round of screening examined 150 knockout candidates in triplicate, and knockouts that at least doubled the total dauer percentage of worms developed on the K-12 control strain were scored as positive (Fig. 1a). Using this approach, we identified 56 mutant *E. coli* strains that robustly enhanced entry into the dauer stage (Table 1). PCR with gene-specific primers was performed to confirm gene deletion in the knockout mutants (Fig. S1A). As negative controls, ten knockout strains that were not identified in the primary screen were randomly chosen and thoroughly tested for dauer formation. These strains did not show any significant alteration in the rate of dauer formation when compared to controls (Fig. S1B).

To identify the biological pathways and cellular functions affected by the bacterial mutants that influenced dauer formation, the candidates were analyzed using PANTHER classification system^20^,^21^. Out of 56 genes, 9 genes are not characterized, and the remaining 47 genes were analyzed for molecular and biological functions (Fig. 1b and Fig. S1C). The following broad molecular functional categories were identified: translation regulator activity (GO:0045182) (4 genes). binding (GO:0005488) (7 genes), structural molecule activity (GO:0005198) (1 gene), catalytic activity (GO:0003824) (17 genes), transporter activity (GO:0005215) (4 genes) (Fig. 1b). Using the PANTHER classification system the cellular site of action of the product of these genes was identified:membrane (GO:0016020), macromolecular complex (GO:0032991), cell part (GO:0044464) and organelle (GO:0043226) (Fig. 1c). Furthermore, the candidate genes were analyzed and assigned functional groups utilizing Clusters of Orthologous Group (COG) terms^22^ and confirmed with MultiFun^23^ and Gene Ontology (GO) classifications^24^. Categorization of the genes revealed the enrichment in the following functional groups: metabolism (~39%); inorganic ion transport and metabolism (~17%); translation, post-translational modification, protein turnover (~12%), and chaperones; cell envelope biogenesis and outer membrane structures (~10%); DNA replication, recombination and repair (~7%); transcription (~5%); and rest other functions (~10%) (Table 1 and Fig. S1D). Furthermore, several of the genes from the screen fell within functional groups and transcriptional units, e.g. operons and regulons (Fig. S1E). Together these findings indicate that the bacterial genes with a broad range of cellular functions and processes can alter *C. elegans* physiology through changes in bacteria derived metabolites or nutrients.

### Phenotypic analyses of bacterial mutants that enhance dauer formation for DAF-16 activation and lifespan extension

**Figure 2:**
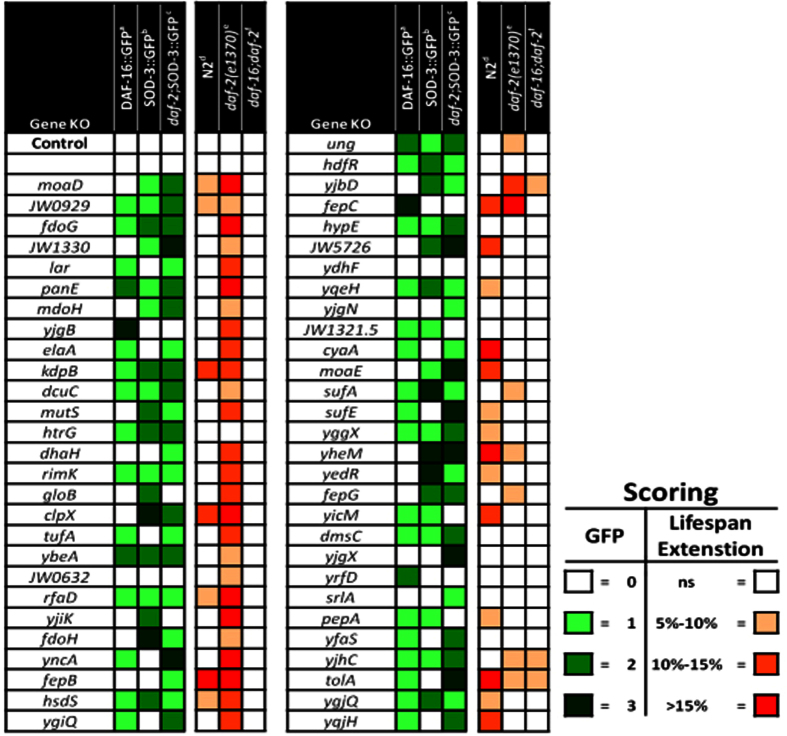
Analysis of DAF-16 activation and lifespan extension in bacterial knockouts that enhance *C. elegans* dauer formation. Heat map showing various phenotypes observed with bacterial mutants (left to right). (**a**) Semi-quantitative analysis of DAF-16 nuclear localization is shown following feeding on listed *E. coli* knockouts. Increased green shading correlates with increasing nuclear localization of DAF-16. (Fig. S2) (**b**) *sod-3p*::*GFP* expression is shown following feeding on listed *E. coli* knockouts. Increased green shading correlates with increasing *sod-3p::GFP* expression. (**c**) *daf-2(e1370*); *sod-3p::GFP* expression is shown following feeding on listed *E. coli* knockouts. Increased green shading correlates with increasing *daf-2(e1370*);*sod-3p::GFP* expression. In each visual marker (**a**–**c**) knockout strains are given a score relative to its K-12 control, which was consistently set at a score of 0 (white shading). (**d**–**f**) Lifespan analysis of respective worm strains following feeding on bacterial knockout strains throughout life. Deeper red shading indicates increased average lifespan compared to K-12 control strain. Representative lifespan curves and lifespan data analysis are shown in Fig. S2 and Table 2 respectively.

The phosphorylation cascade elicited by an activated ILS negatively regulates DAF-16 by sequestering it in the cytoplasm. Conversely, reduced ILS signaling leads to diminished phosphorylation of DAF-16, thereby allowing it to enter the nucleus and modify gene expression^25^,^26^. Given the effects of the bacterial mutants on dauer formation in the *daf-2* mutant, we examined whether they would also impact DAF-16 in adult animals. A majority (∼63%) of the bacterial mutants promoted DAF-16 nuclear localization in a worm strain expressing a DAF-16::GFP (daf-16(mgDf47)) fusion construct (Fig. 2 and Fig. S2A). Once DAF-16 enters the nucleus it can modulate the transcription of its targets, such as the stress-responsive superoxide dismutase, SOD-3^27^. Consistently, ~64% of the bacterial mutants also increased *SOD-3p::GFP* expression in an N2 control background, while ~80% of the bacterial knockouts increased *sod-3* expression in the *daf-2(e1370*) background (Fig. 2 and Fig. S2A). No significant effects on *sod-3* expression were noted in the 10 negative controls used previously, compared to animals fed control bacteria (data not shown). Lastly, of the 35 bacterial candidates that showed DAF-16 nuclear localization, 26 induced *sod-3p::GFP* in N2 background, while 32 induced GFP in the *daf-2;sod-3p::GFP* strain (Fig. 2). Taken together these data suggest that the majority of *E. coli* mutants from the screen stimulate DAF-16 translocation and/or subsequent regulation of its transcriptional targets in both the *daf-2* mutant and control (N2) worms.

In addition to regulating dauer entry during development, DAF-16 exhibits transcriptional control over genes determining longevity in the adult worm^27^,^28^. Thus, we assessed the effect of each bacterial strain identified in the screen on the survival of N2, *daf-2*, and *daf-16*; *daf-2* strains. Feeding several of the *E. coli* knockout strains resulted in increased lifespan in both the *daf-2* and control (N2) background. However, ~61% of the bacterial mutants significantly extended the average lifespan of *daf-2* mutants compared to ~38% in the control N2 strain. (Fig. 2, Table 2 and Fig. S2B). These observations were similar to the results obtained with the *sod-3::gfp* expression (Fig. 2), which can potentially be explained as the screen was carried out in a sensitized *daf-2* mutant background. Although most of the knockout bacteria appear to modulate lifespan in a *daf-16*-dependent manner (Fig. 2 and Table 2), some bacterial mutants (e.g. *tolA*, and *yjbD*) extended lifespan in *daf-16;daf-2* mutant worms, indicating that a genetically modified diet may also affect longevity by *daf-16*-independent mechanisms.

Several of the identified bacterial genes appear to have highly directed roles in micronutrient or metabolite production or transport (Table 1 and Fig. S1E). For example, *fepB, C,* and *G* have roles in the iron import system. *dhaH* has an enzymatic role in the production of dihydroxyacetone phosphate, while *srlA, yjhc,* and *mdoH* are involved in the production of sorbitol, N-acetylneuraminic acid (sialic acid), and periplasmic glucans, respectively. One of the largest lifespan extension was observed with *cyaA*, which encodes adenylate cyclase that catalyzes the conversion of ATP to Adenosine 3′,5′-cyclic monophosphate (cAMP) and pyrophosphate^15^. In bacteria, intracellular cAMP signaling modulates the expression of catabolic proteins with biosynthetic and ribosomal proteins according to cellular metabolic needs during exponential growth^29^. We further characterized *cyaA* mutant bacteria to determine the role of bacterial cAMP in modulating insulin signaling, dauer formation and lifespan in *C. elegans*.

### cAMP modulates lifespan and dauer formation in *C.elegans*

**Figure 3:**
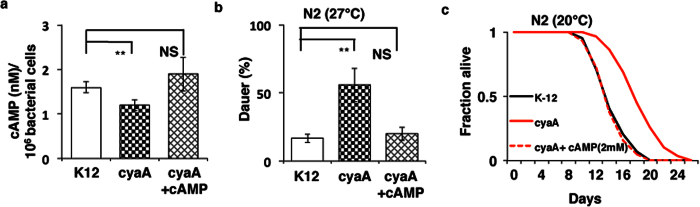
Feeding bacterial *cyaA* mutants modulates lifespan and dauer formation in *C. elegans*. (**a**) The amount of cAMP in K-12 (control), mutant *cyaA* bacteria, *cyaA* mutant bacteria supplemented with cAMP (2 mM), (**b**) Bacterial knockout of *cyaA* enhances the dauer formation in N2 strain. Control (N2) animals fed on K-12 (control) bacteria, mutant *cyaA* bacteria and *cyaA* mutant bacteria supplemented with cAMP (2 mM) on minimal media agar plate. Data is represented as mean percent ± SD of greater than 3 biological replicates, n > 200. (**c**) Feeding *E. coli cyaA* mutants extends the lifespan of N2 strain in *C. elegans*. ***P < 0.0001.

We examined whether the effect of *cyaA* mutant bacteria on dauer formation was a consequence of reduced levels of cAMP. We confirmed that cyaA gene is disrupted (Fig. S1) and cAMP levels were indeed reduced in *cyaA* mutant bacteria (Fig. 3a). Next, we found that supplementation with 2 mM exogenous cAMP could restore levels back to those observed in K12 bacteria (Fig. 3a). Also, we did not see any significant difference in the bacterial growth curves between cyaA mutant bacteria as compared K-12 bacteria under minimal media conditions (Fig. S3A). The uptake of cAMP has been shown to be variable in bacteria and exhibits saturation kinetics based on the culture conditions^30^. Also, worms fed on *cyaA* mutant bacteria did not display any apparent adverse effects on worm physiology, as we did not observe any significant change in physiological parameters such as body size, lawn leaving behavior or pharyngeal pumping in control animals (N2) fed on *cyaA* mutant bacteria (Fig. S3B–G). Furthermore, bacterial choice assays suggested that the N2 worms preferred K-12 control bacteria to *cyaA* and other mutant bacteria that enhance dauer formation (Fig. S3H).

Higher temperature has also been shown to enhance dauer formation^31^. We observed that at 27 °C, ~56% of N2 animals grown on *cyaA* bacteria formed dauers, compared with only ~20% dauers when grown on K12 bacteria (Fig. 3b, Table S1 and Fig. S4A). However, the dauer enhancement was rescued when *cyaA* bacteria were supplemented with 2 mM cAMP. Next, we performed a dauer assay with *daf-2(m41*) mutant worms that make almost 100% dauer at semi-permissive temperature. We observed a ~20% inhibition of dauer formation on the addition of cAMP (2 mM) in *daf-2(m41*) mutant worms (Fig. S4B). In lifespan experiments, N2 worms fed on cyaA mutant bacteria displayed a ~35% increase in lifespan. The addition of 2 mM cAMP to *cyaA* bacteria significantly suppressed the lifespan extension observed in the *cyaA* mutant bacteria alone (Fig. 3c, Table S2 and Fig. S4C). Thus both the dauer and lifespan effects of *cyaA* mutant bacteria could be rescued by exogenous addition of cAMP.

### Bacterial *cyaA* mutant modulates dauer formation and lifespan through TGF-β signaling and DAF-16 activation

**Figure 4:**
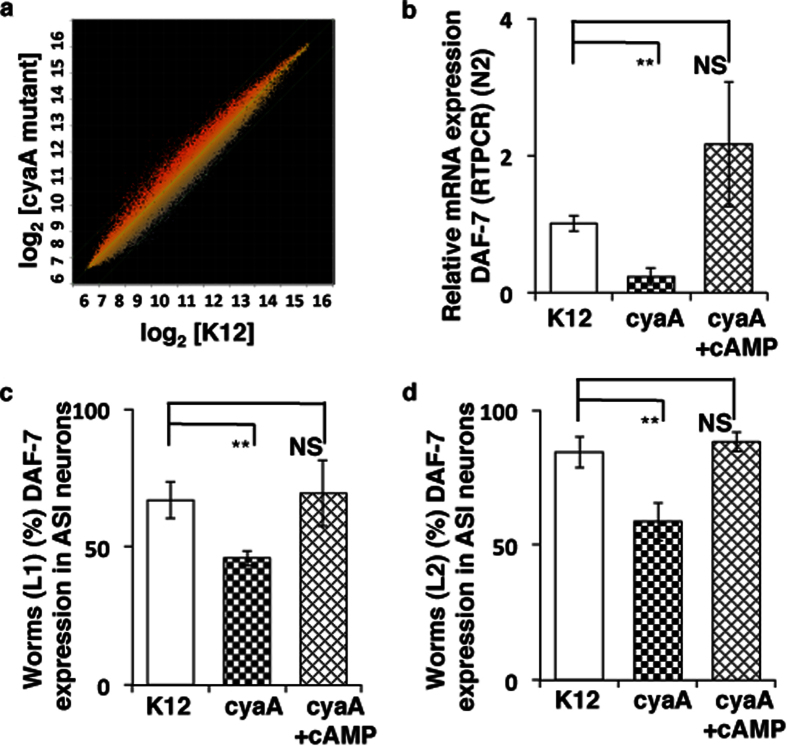
Feeding bacterial *cyaA* mutants alters DAF-7 expression in *C. elegans*. (**a**) A scatter plot of log2 transcript probe intensities for *daf-2(e1370*) versus N2, and K-12 (control) versus *cyaA* fed control worm comparisons. Genes which show only p-values of <0.05 and fold changes >1.3 and <0.8 were shortlisted. (**b**) Relative expression of *daf-7* mRNA in N2 animals on feeding K-12(control), *cyaA* mutant bacteria, and *cyaA* mutant bacteria supplemented with cAMP(2 mM). (**c,d**) cAMP induces the expression of DAF-7 in ASI neuron. The fraction of animals show DAF-7 expression at L1 and L2 stage on feeding K-12 (control), cyaA mutant bacteria, and cyaA mutant bacteria supplemented with cAMP (2 mM). The data is represented as percent mean ± S.D. of greater than 3 biological replicates, n > 200, **P < 0.0001.

To investigate the signaling mechanism by which *cyaA* mutant bacteria modulates host *C. elegans* physiology to enhance dauer formation and extend lifespan, we examined the gene expression changes modulated by *cyaA* bacteria in control animals, using microarray profiling in adult animals fed *cyaA* bacteria and K12 bacteria (Fig. 4a and Table S3). Using GoMiner high-throughput analysis, we discovered multiple enriched biological processes within our data set (Fig. S5A). Of particular interest to us was the significant parent-child GO-term set of aging, multicellular organismal aging, and determination of adult lifespan. Comparison of changes in gene expression in the *daf-*2 background from previously published^32^ and our own data sets with transcriptionally altered genes in *cyaA*, showed considerable overlap (Fig. S5B,C). Collectively, these data suggest that feeding *cyaA* mutant *E. coli* activates DAF-16 to enhance dauer formation and extend lifespan.

We then examined the mechanism of activation of DAF-16 by measuring the expression of genes upstream of DAF-16 known to be involved in dauer formation. We observed that *daf-7,* a gene encoding a TGF-β-like ligand, was down-regulated in control worms grown on *cyaA* bacteria. To confirm this, we examined the expression of mRNA of *daf-7* by RT-PCR in one-day adult control animals on *cyaA* mutant bacteria, compared with animals fed with K-12 or *cyaA* supplemented with cAMP (2 mM). DAF-7 expression was significantly reduced (~50%) on feeding animals with *cyaA* mutant bacteria and was rescued by addition of cAMP (2 mM) to the bacterial lawns (Fig. 4b). We also examined DAF-7::GFP expression in the ASI chemosensory neuron in L1 and L2 when fed with *cyaA* mutant bacteria. We observed that *cyaA* mutant bacteria inhibited the expression DAF-7::GFP in L1 and L2 stage in comparison to K-12 bacteria (Fig. 4c,d). Conversely, supplementation with 2 mM exogenous cAMP to *cyaA* mutant bacteria increased the expression of DAF-7 in a fraction of L1 and L2 animals (Fig. 4c,d). These results suggest that cAMP levels in the bacteria can modulate the expression of DAF-7 in worms, which influences dauer formation and insulin signaling^33^.

**Figure 5:**
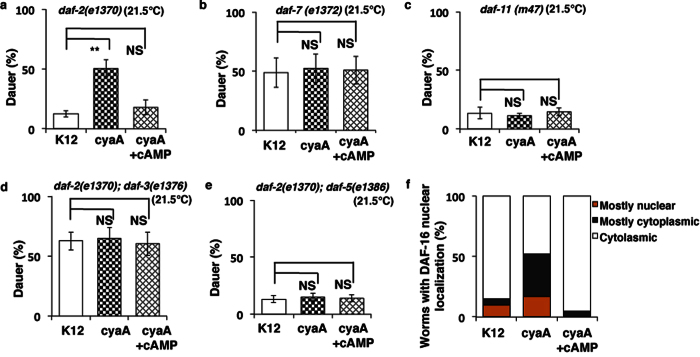
Bacterial *cyaA* mutants affect dauer formation through TGF-β pathway and DAF-16. (**a–e**) Dauer formation of *daf-2(e1370), daf-7(e1372), daf-11(m47) and daf-2(e1370); daf-3(e1376), daf-2(e1370); daf-5(e1386*) animals on feeding K-12 (control), *cyaA* mutant bacteria, and *cyaA* mutant bacteria supplemented with cAMP (2 mM). (**f**) DAF-16::GFP worms in the N2 background on feeding K-12 (control), *cyaA* mutant bacteria, and *cyaA* mutant bacteria supplemented with cAMP(2 mM). Quantification of DAF-16::GFP localization on feeding K-12 (control), *cyaA* mutant bacteria, and *cyaA* mutant bacteria supplemented with cAMP(2 mM). In each case, the data is represented as mean percent ± S.D of three replicates, **P < 0.0001, n > 200.

To further examine how *cyaA* mutant bacteria and cAMP modulate dauer formation we carried out epistasis analysis on *C. elegans* mutants using mutants in the three major pathways involved in dauer formation^16^,^17^,^34^. As shown in Fig. 3 dauer entry in *daf-2(1370*) mutants, was significantly increased when fed on *cyaA* mutant bacteria but was reversed by addition of exogenous cAMP. However, neither the *daf-7(e1372*) mutant, which is defective in TGF-β signaling nor the *daf-11(m47*) mutant, which has defective cyclic GMP signaling and is involved in DAF-7 secretion, showed any enhancement of dauer formation when fed on *cyaA* mutant bacteria (Fig. 5a–c and Table S1). This data supports the hypothesis that reduced bacterial cAMP enhances dauer formation by a similar mechanism as inhibition cGMP and TGF-β signaling pathways in worms.

The constitutive dauer formation phenotype of *daf-7(e1372*), but not *daf-2(e1370*), is suppressed by mutations in *daf-7* downstream factors *daf-3* and *daf-5*^2^. The introduction of either *daf-3(e1376*) or *daf-5(e1386*) into the *daf-2(e1370*) background prevented *cyaA* mutant bacteria from enhancing dauer formation (Fig. 5d–e and Table S1). This suggests that *cyaA* mutant bacteria acts through TGF-β signaling to activate DAF-16, and enhancing dauer formation. We confirmed our finding from the screen that worms fed on *cyaA* mutant bacteria show increased nuclear localization of DAF-16 (Fig. 5f). Furthermore, not only was the nuclear localization of DAF-16::GFP enhanced on *cyaA* mutant bacteria compared with the K12 control, but it was also reversed upon supplementation of bacterial lawns with exogenous cAMP (Fig. 5f).

**Figure 6:**
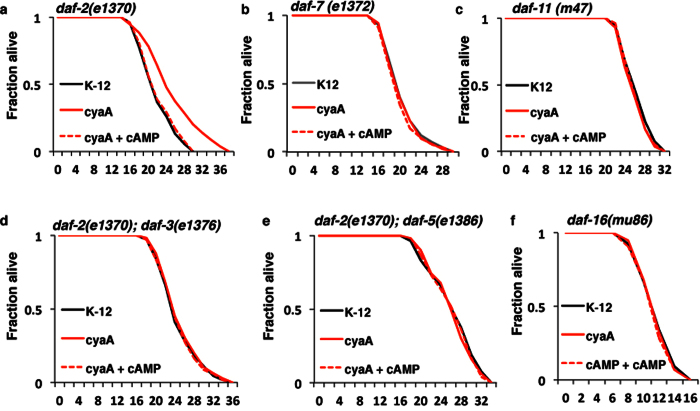
Bacterial *cyaA* mutants affect lifespan through TGF-β pathway and DAF-16. (**a–f**) Kaplan–Meier survival curves of synchronously aging hermaphrodite worms: *daf-2(e1370), daf-7(e1372), daf-11(m47*), *daf-2(e1370); daf-3(e1376), daf-2(e1370); daf-5(e1386*) and *daf-16(mu86*) animals fed on K-12 (control), *cyaA* mutant bacteria, and *cyaA* mutant bacteria supplemented with cAMP (2 mM). n = 100, **p < 0.01; average ± std. dev (n = 3).

Conserved molecular pathways that regulate dauer formation like Insulin/IGF-1 signaling (IIS) and TGF-β signaling pathways also regulate longevity. This also appears to be the mechanism by which *cyaA* bacteria extend lifespan, since *cyaA* bacteria did not extend lifespan in either the *daf-7(e1372*) or the *daf-11(m47*) mutant backgrounds, nor did it extend lifespan in *daf-2(e1370); daf-3(e1376*) or *daf-2(e1370); daf-5(e1386*) double mutant backgrounds (Fig. 6a–f and Table S2). Together, these data indicates that reduced bacterial cAMP acts via the TGF-β signaling pathway to enhance DAF-16 resulting in increased dauer formation and lifespan.

## Discussion

The type of bacterial food source modulates *C. elegans* development and lifespan^35^,^36^,^37^,^38^,^39^,^40^,^41^,^42^,^43^,^44^. In this study, we have screened for bacterial mutants that regulate dauer formation in *C. elegans* to understand the role of bacterial genes in modulating nutrient sensing pathways. We used the Keio mutant *E. coli* library^15^ to identify individual gene knockout mutants that affect bacterial signals that enhance dauer formation in *C. elegans*. Genes mutated among the bacterial mutants identified in the dauer screen have varied functions (Fig. 1b) including, metabolism, translation, biogenesis of membrane and DNA replication (Fig. 1b and Table 1). These results suggest that bacteria act not only as a food source but also provide signals that influence nutrient signaling pathways in *C. elegans*. A better understanding of *E. coli* genes that influence these signaling pathways could give us a better understanding of complex host-bacterial relationships in other species.

In the absence of bacterial food, *C. elegans* show reduced rates of growth and progeny production and lifespan extension^45^. These observations can be explained by the idea that worms subjected to suboptimal nutrition or the absence of toxic or pathogenic components of bacteria, extend lifespan. Previously, different bacterial diets^10^, and bacterial metabolites like folate^36^ and fatty acids^14^ have been proposed to modulate lifespan. Our results identify several bacterial genes that are involved in the production or transport of various downstream metabolites to regulate dauer formation and lifespan in worms. Furthermore, we observed that the worms fed on dead bacteria show increase in dauer formation (Fig. S5D), which suggests that viable secondary metabolites can have a profound effect on bacterial physiology that can modulate dauer formation in worms. To further examine if the cAMP-mediated dauer rescue was due to the direct uptake of cAMP by the worm, or an indirect effect on bacterial metabolism, we grew worms on UV-killed bacteria supplemented with cAMP. In these experiments, we found that there was an increase in basal dauer formation in both the K12 and *cyaA* controls, but that the addition of cAMP to UV-killed *cyaA* bacteria did not significantly reduce the dauer entry phenotype (Fig. S5D). These experiments suggest that action of cAMP requires live bacteria to mediate its effects on dauer formation.

There are several pathways involved in dauer formation, including guanylyl cyclase, ILS, and TGF-β^2^. A critical downstream factor of some of these pathways is DAF-16 which has been shown to integrate inputs from various upstream dauer formation pathways^2^,^46^. Most of the bacterial mutants that enhance dauer formation from our screen do not extend lifespan in *daf-16* null mutant animals and enhance nuclear localization of DAF-16 to a variable extent (Fig. 2). These bacterial mutants also enhance the expression of SOD-3, a direct target of DAF-16^47^,^48^. These data suggest that several but not all of these bacterial mutants require DAF-16/FOXO for increased longevity and improved health span, implicating bacterial metabolites in modulating nutrient sensing signaling pathways^49^.

**Figure 7:**
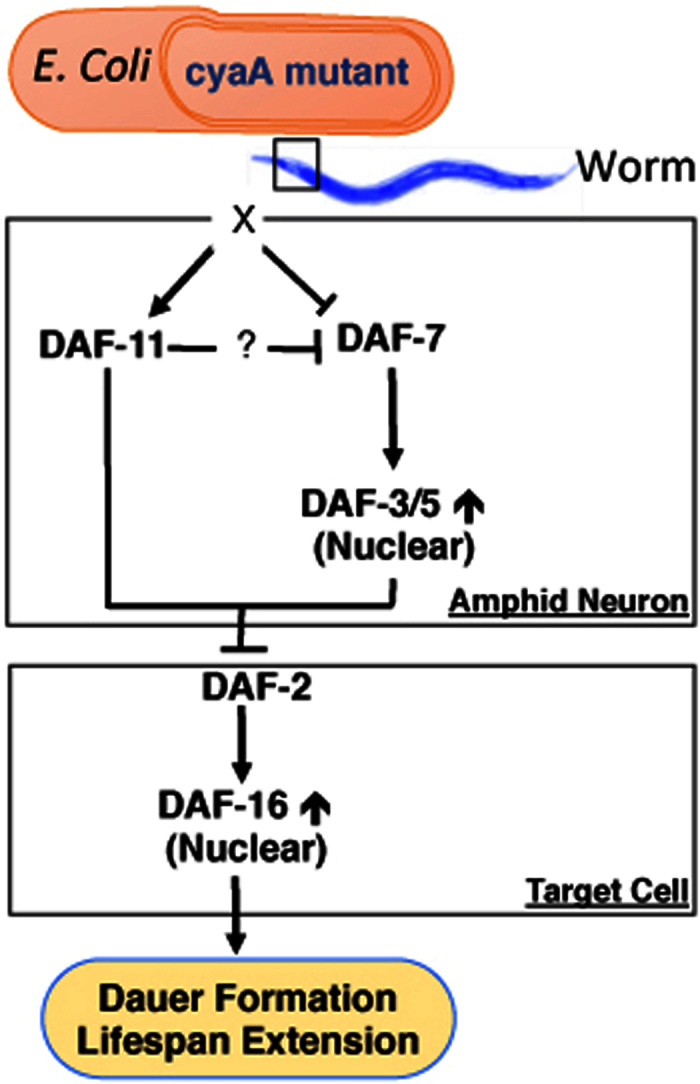
Model of how bacterial cAMP signaling regulates dauer formation and lifespan in worms. Lack of bacterial cAMP inhibits the expression of DAF-7, resulting in the inhibition of DAF-3/5. This leads to the suppression of IGF-1 pathway in animals, and enhances the dauer formation and lifespan extension in DAF-16/FOXO dependent manner.

We also identified that bacteria deficient in *cyaA* inhibit TGF-β signaling to enhance DAF-16/FOXO to enhance dauer formation and lifespan. Dauer assays using *daf-7* and *daf-11* null mutants suggests that the effects of feeding on the *cyaA* mutant bacteria on dauer formation require TGF-β and the guanylyl cyclase (DAF-11) expression^2^,^27^,^46^. DAF-11 is localized primarily in the ciliary endings of some of the amphid neurons and integrates environmental signals from G-protein coupled receptors (GPCR)^50^. As mentioned before, ILPs and DAF-7 are expressed and released primarily by neurons and ASI neurosensory cells, respectively^16^. Thus we speculate that *cyaA* mutant bacteria differentially impact these sensory neurons to regulate changes in dauer formation and lifespan. We speculate that this regulation may be carried out by reducing cAMP levels produced by the *E. coli*, resulting in changes in secondary metabolites or cAMP itself^29^. Based on our observation it appears that feeding *cyaA* mutant bacteria modulates DAF-11 signaling, decreasing DAF-7 neuronal expression (Fig. 7). Further studies are required to uncover the cross talk between *cyaA* dependent bacterial products on *C. elegans* signaling pathways.

Bacterial products as part of the gut microbiome affect many biological processes such as metabolism, longevity, and health span in mammals^16^,^35^. The function of some of these bacteria has been well described in several human diseases including obesity, liver diseases, metabolic syndrome, autoimmune disorders, and diabetes^51^,^52^,^53^. Some of these microbiome related products modulate diverse host metabolic activities resulting in pathologies related to insulin sensitivity^54^,^55^. However, the mechanisms by which individual bacterial gene products interact with the host to modulate insulin signaling pathways are not known^49^. Gut microbiome’s influence on biological processes has been observed across different species from insects to mammals. In this study, we describe how mono-association of bacterial mutants with *C. elegans* can help decipher how specific bacterial signals modulate host physiology. Thus interaction between bacteria and *C. elegans* can be used to understand conserved signals that are likely to play a role in host-microbiome interactions in humans which may influence diseases like Type II diabetes and obesity.

## Methods

### *C. elegans* strains and growth conditions

All strains were obtained from the *Caenorhabditis* Genetics Center (CGC). The strains obtained from the CGC were outcrossed 6 times to N2 (control) worms. Strains used in this manuscript from CGC are: CB1370 *daf-2(e1370) III,* CB1372 *daf-7(e1372) III,* DR47 *daf-11(m47) V,* CF1038 *daf-16(mu86) I,* HT1608 *daf-2(e1370) III; daf-3(e1376) X,*CF1553 *muIs84 [(pAD76) sod-3p::GFP* + *rol-6(su1006*)], DR1624 *daf-5(e1386) II; daf-2(e1370) III,* CF1139 muIs61 [(pKL78) daf16::GFP + rol-6(su1006)], DR1564 *daf-2(m41) III,* FK181 *ksIs2 [daf-7p::GFP* + *rol-6(su1006)], TM151 sod-2(sj173) I; daf-2(e1370) III; sod-3(sj134) X* and PR678 *tax-4(p678) III; daf-11(m47) V*^56^. Strains were maintained on nematode growth media plates at 20 °C seeded with *E. coli (K12*) bacteria grown in Luria broth. For egg collection, gravid adults were soaked in hypochlorite solution as described previously^57^.

### *E. coli* strains

*E. coli* mutant strains, including the parent *K12* BW25113 strain, were obtained from the Keio mutant collection^15^. The strain library was shipped and stored as a frozen stock in 96-well plates. Stock plates were pin-replicated to produce a working copy plate containing Luria Broth (LB) and 25 μg/ml kanamycin. For assays used in this study, *E. coli* strains were stab cultured and grown overnight with shaking at 37 °C in minimal media as described below. Overnight cultures were seeded on minimal media plates and dried at 37 °C for 12 hours prior to transfer of *C. elegans*.

### Media

Luria broth (LB) and nematode growth media (NGM) were used to maintain stock cultures of *E. coli* and *C. elegans* strains, respectively. Minimal media was used for assays in this study as described previously^58^. Liquid media for bacterial growth contained: 0.05 M NaCl, 0.04 M NH_4_Cl, 0.01 M CaCl_2_, 0.025 M phosphate buffer, 0.4% glucose, 1 μg /mL thiamine, and 0.001 M MgSO_4_. Minimal media plates for subsequent *C. elegans* experiments contained: 2% agar, 0.05 M NaCl, 0.04 M NH_4_Cl, 0.001 M CaCl_2_, 1 μg/mL cholesterol, 0.025 M phosphate buffer, 0.4% glucose, 1 μg/mL thiamine, and 0.001 M MgSO_4._ All compounds supplemented to the agar plates for rescue experiments were purchased from Sigma-Aldrich.

### PCR confirmation

Bacterial strains for kanamycin cassette confirmation were streaked onto LB plates containing 25 μg/ml kanamycin. Strains were colony purified, and genomic PCR was performed. The primer sets were designed for individual knockout strains around the outside of the target gene. The forward primer was designed to be within 200 bp upstream of the 5′ end, and the reverse primer was within 200 bp downstream of the 3′ end of the target gene. The thermal cycler parameters were as follows: initial 5 min at 94 °C, then 25 cycles of 30 s at 94 °C, 10 s at 56 °C, 1 min at 72 °C, and final 5 min at 72 °C. Products were run on an agarose gel and amplified products were examined for appropriate length. Representative list of primers (5′ to 3′).


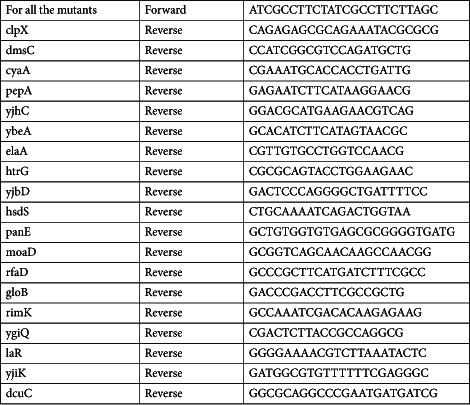


### RT-PCR validation

Quantitative real-time PCR was carried out using SYBR Green I (Sigma Aldrich) assay reagents to verify gene expression profiles. 200 ηg of total RNA were reverse transcribed to cDNA and the real-time PCR reactions were performed on a 7500 Fast System Real-Time PCR cycler (Applied Biosystems, Foster City, CA), according to the manufacturer’s instructions. The gene expression fold changes between different treatment groups were calculated using the delta Ct method^59^.


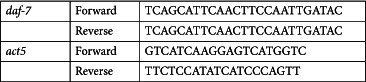


### Dauer assay

#### Dauer assay using bacterial mutants

Bacterial strains were grown to stationary phase in minimal medium prior to being seeded on minimal media agar. Mutant and control *E. coli* plates were left to grow overnight at 37 °C. Eggs were placed on plates containing the *E. coli* mutants. Plates were incubated at 21.5 °C for 4–5 days prior to assessing dauer or non-dauer state worms. Worm larval states were analyzed using a dissecting microscope and manually counted.

### Measurement of cAMP

cAMP levels from bacterial strains and worm strains were measured using the cAMP- Glo TM assay kit purchased from Promega (Madison, WI). For bacteria, 5 ml cultures of K-12, cyaA were grown with or without the presence of cAMP (2 mM) in minimal media at 37 °C for overnight. Cells were counted in 50 μl bacterial culture and were sonicated for complete lysis, and 5 μl of the lysate was used to measure cAMP. The number of bacteria was counted with standard bacterial colony count protocol and the amount of cAMP in different bacteria was expressed per 10^6^ bacterial cells. PBS for complete lysis. 5 μl sample of the lysate was used for cAMP measurement, and the amount of cAMP was expressed per 200 animals.

### Fluorescence Microscopy

Age-synchronized L1-stage animals carrying DAF-16::GFP integrated transgenes (*daf-16(mgDf47*) I) were placed on plates seeded with overnight cultures of bacterial strains as described above. Animals were grown at 15 °C for 2–3 days and then moved to 21.5 °C for one day unless otherwise noted. The fluorescent images were captured using reflected light fluorescence microscopy (Olympus IX3) and the images were then processed for densitometry analysis using ImageJ.

### Lifespan assay

Late L4 larvae growing at 15 °C were transferred to fresh minimal media plates with FUdR (5 μg/ml) added to the bacterial lawn as indicated in the results. The first day of adulthood is day 1 in survival curves. Animals were scored as alive, dead or lost every other day. Animals that failed to display touch-provoked movement were scored as dead. Animals that died from causes other than aging, such as sticking to the plate walls, internal hatching or bursting in the vulval region, were scored as lost. Animals were transferred to fresh plates and fresh *E. coli* every 2 days with cAMP added to have final concentration 2 mM. Plates were dried for 15 minutes at 20 °C before the worms were transferred. All lifespan experiments were performed at 21.5 °C. Survival curves were plotted, and statistical analyses (log-rank tests) were performed using Prism 4 software (Graphpad Software, Inc., San Diego, CA, USA). Statistical significance between lifespan curves was determined by p-value of <0.05 and a mean lifespan of >5% difference compared to the control mean as described before^32^,^60^.

### Behavioral assays: Pharyngeal pumping and body bends

Worms were observed using stereomicroscope at 40x magnification. Pumps per minute were counted as described previously^61^. Body bends per minute were counted as described previously^62^.

### Bacterial choice assay

Age-synchronized day 1 adult worms were used. Minimal media plates were used for this assay: 2% agar, 0.05 M NaCl, 0.04 M NH_4_Cl, 0.001 M CaCl_2_, 5 μg/mL cholesterol, 0.025 M phosphate buffer, 0.4% glucose, 1 μg/mL thiamine, and 0.001 M MgSO_4._ Overnight bacterial culture with OD 600 nm of 2.0 was spotted (30 μl) equidistant from the center of the plate and dried for 15 minutes at 37 °C. Both K12 and mutant bacteria had approximately the same cellular density: K12 bacteria at 0.8 × 10^7^ ± 1.2 × 10^7^ colony-forming units (cfu) per ml and dmsC mutant bacteria at 0.7 × 10^7^ ± 1.3 × 10^7^ colony-forming units (cfu) per ml. Worms were washed and prepared as previously described^63^. The worms were placed in the center of the plate, and positions of the worms were observed using stereomicroscope and images taken at 15 minutes interval. Percentage of worms at each lawn at (1 hr) were plotted as a histogram.

### *C. elegans* feeding behaviour assays

Feeding behavior was assessed based on bacteria depletion as described before^64^. Minimal media plates were used for this assay: 2% agar, 0.05 M NaCl, 0.04 M NH_4_Cl, 0.001 M CaCl_2_, 5 μg/mL cholesterol, 0.025 M phosphate buffer, 0.4% glucose, 1 μg/mL thiamine, and 0.001 M MgSO_4._ Overnight bacterial culture with OD 600 nm of 0.8 was spotted (60 μl) in center of the plate with streotomycin (300 ng/ml), carbenicillin & Kanamycin (50 μg/ml), and FUdR (5 μg/ml) and the plates were dried for 15 minutes at 37 °C. Age synchronized late L4 worms (n = 60) were washed and prepared as previously described^63^,^64^ and introduced on the plates. The worms were left on the plates overnight at 20 °C. Control plates with bacteria spotted as described above with no worms were also incubated at 20 °C overnight. After 24 hrs the bacteria was washed off the plates and checked for OD at 600 nm.

### Lawn leaving assay

Age-synchronized day 1 adult animals (n = 20 per plate) were transferred to bacterial lawns cultured under conditions described above. Assay was performed for 5 minutes and lawn leaving events were manually counted by observing under the microscope and averaged to lawn leaving events per minute as previously described^65^.

### Statistical Analysis

Microarray data analysis was performed using SPSS Professional Edition software (IBM). Descriptive statistics including mean and s.e.m. along with one-way ANOVAs followed by multiple comparison tests and two-tailed T-test were used to determine significant differences. P < 0.05 was considered significant as described before^32^,^66^. For lifespan assays, log-rank tests were performed by the Prism 4 software as described before^32^,^60^. Pearson product-moment correlation coefficient was calculated to measure the correlation between data sets.

## Additional Information

**How to cite this article**: Khanna, A. *et al*. A genome-wide screen of bacterial mutants that enhance dauer formation in *C. elegans. Sci. Rep.* **6**, 38764; doi: 10.1038/srep38764 (2016).

**Publisher's note:** Springer Nature remains neutral with regard to jurisdictional claims in published maps and institutional affiliations.

## Supplementary Material

Supplementary Data

Supplementary Table 3

## Figures and Tables

**Table 1:** List of bacterial mutants that enhance dauer formation.

Gene Knockout	Brief Description	Func. Group	Mean(%)^a^	±SE^b^	n^c^	*p-Value*^d^
K-12	Control	/	12.6	1.5	231	/
moaD	molybdopterin synthase, small subunit	MET	81.9	3.2	117	<0.0001
JW09292	zapC: cell division factor, localises to the cytokinetics ring	OTR	79.0	2.6	309	<0.0001
fdoG	α subunit of formate dehydrogenase O	MET	77.7	0.9	63	<0.0001
JW1330	abgT; predicted cryptic aminobenzoyl-glutamate transporter	MET	72.1	3.5	222	<0.0001
Lar	Rac prophae: restriction alleviation protein	OTR	70.2	2.0	145	<0.0001
panE	2-dehydropantoate reductase, NADPH-specific	MET	68.6	3.8	98	<0.0001
mdoH	glucan biosynthesis: glycosyl transferase	CEB	67.5	7.4	96.0	0.00
yjgB	predicted alcohol dehydrogenase, Zn-dependent and NAD(P)-binding	UNK	67.5	3.3	180	<0.0001
elaA	predicted acyltransferase, Zn- dependent and NAD(P)-binding	UNK	66.7	3.2	207	<0.0001
kdpB	potassium translocating ATPase, subunit B	ITM	66.4	17.5	103	0.04
dcuC	anaerobic C4-dicarboxylate transport	MET	65.2	1.6	186	<0.0001
mutS	methyl-directed mismatch repair protein	DNA	64.4	3.6	209	<0.0001
htrG	predicted signal transduction protein (SH3 domain)	UNK	64.3	5.4	194	<0.0001
dhaH	fused predicted dihydroxyacetone-specific PTS enzymes: HPr component	MET	63.4	11.4	63	0.01
rimK	ribosomal protein S6 modification protein	TRN	62.8	3.3	72	<0.0001
gloB	predicted hydroxyacylglutathione hydrolase	MET	62.6	8.6	95.0	0.00
clpX	ATPase and specificity subunit of ClpX-ClcP ATP-dependent serine protease	TRN	62.4	2.3	175	<0.0001
tufa	protein chain elongation factor EF-Tu (duplicate of tuFB)	TRN	60.7	3.0	69	<0.0001
ybeA	23S rRNA mψ1915 methyltranferase	OTR	60.2	7.6	250	0.00
JW0632	ybeB; predicted protein	UNK	58.7	6.4	190	0.00
rfaD	ADP-L-glycero-D-mannoheptose-6-epimerase	CEB	58.3	5.0	146	<0.0001
yjiK	conserved protein	UNK	56.4	5.4	151	0.00
fdoH	formate dehydrogenase-O, Fe-S subunit	MET	56.0	10.7	65	0.02
yncA	predicted acyltransferase with acyl-CoA N-acyltransferase domain	CEB	55.8	9.5	135	0.01
fepB	iron-enterbactin transporter subunit	ITM	53.2	10.3	309	0.01
hsdS	specificity detereminant for hsdM and hsdR	DNA	52.3	6.6	188	0.00
ygiQ	conserved protein	UNK	51.0	9.2	63	0.01
Ung	iron-enterbactin transporter subunit	DNA	50.6	11.6	70	0.03
hdfR	DNA-binding transcriptional regulator	TRX	50.4	16.3	200	0.08
yjbD	conserved protein	UNK	49.2	4.0	180	0.00
fepC	iron-enterbactin transporter subunit	ITM	47.8	4.9	142	0.00
hypE	carbomoyl phosphate phosphatase, hydrogenase 3 maturation protein	TRN	45.2	7.4	105	0.01
JW5726	phnl, phnH; carbon-phosphorus lyase complex subunit	ITM	43.5	4.2	112	0.00
ydhF	predicted oxidoreductase	UNK	43.1	6.0	145	0.01
yqeH	conserved protein with bipartite regulator domain	TRX	42.1	4.4	117	0.00
yjgN	conserved inner membrane protein	UNK	41.4	4.8	97	0.00
JW1321.5	Unknown	UNK	40.0	5.7	236	0.01
cyaA	adenylate cyclase	OTR	39.6	7.5	66.0	0.02
moaE	molybdopterin synthase, small subunit	MET	38.3	7.7	82	0.03
sufA	Fe-S cluster assembly protein	MET	37.6	5.1	229	0.01
sufE	sulphur acceptor protein	MET	36.6	5.1	211	0.01
yggX	protein that protects iron-sulfur proteins against oxidative damage	TRN	36.1	8.1	352	0.05
yheM	predicted intracellular sulfur oxidation protein	ITM	36.0	2.8	208	0.00
yedR	predicted inner membrane protein	UNK	33.5	8.1	294	0.07
fepG	iron-enterbactin transporter subunit	ITM	32.3	6.8	195	0.03
yicM	purine ribonucleoside exporter	MET	32.2	9.0	187	0.10
dmsC	dimethyl sulfoxide reductase, anaerobic, subunit C	MET	32.1	1.8	111	0.00
yjgX	KpLE2 phage-like element; predicted protein (pseudogene)	UNK	31.8	4.8	181	0.02
yrfD	predicted pilus assembly protein	MET	31.6	4.9	195	0.02
srlA	glucitol/sorbitol -specific enzyme IIC component of PTS	MET	30.6	4.0	323	0.01
pepA	aminopeptidase A, a cyteinylglycinase	MET	29.8	2.6	216	0.00
yfaS	predicted protein (pseudogene)	UNK	29.7	4.5	223	<0.0001
Yjhc	KpLE2 phage-like element; predicted oxidoreducatse	UNK	29.7	1.6	247.0	<0.001
tolA	membrane anchored protein involved in colicin uptake	CEB	29.2	3.8	186	0.02
ygjQ	predicted thioredoxin-like	UNK	28.6	1.7	252	0.00
yqjH	predicted siderophore interacting protein	ITM	28.3	1.0	367	<0.0001

*E. coli* knockouts were identified as portrayed in Fig. 1a and functional groups were delineated as described in text and Fig. 1b. Brief descriptions were adapted from EcoCyc. Functional codes were abbreviated as follows: MET, metabolism; ITM, inorganic ion transport and metabolism; TRN, translation, posttranslational modification, protein turnover, chaperones; CEB, cell envelope biogenesis, outer membrane; DNA, DNA replication, recombination and repair; TRX, transcription; OTR, other; UNK, unknown.

^a^The mean percentage of dauer staged animals.

^b^The standard error of the mean in percent.

^c^The total number of individuals scored (includes all replicates).

^d^The *p*-value for a student’s two-tailed t-test comparing respective *E. coli* gene knockout strain to control strain.

**Table 2:** Lifespans of *C. elegans* strains fed on different bacterial mutants.

Gene Knockout	N2				*daf-2(e1370*)				*daf-16(mu86); daf-2(e1370*)			
	Mean^a^	Change^b^	n^c^	*p-Value*^d^	Mean^a^	change^b^	n^c^	*p-Value*^d^	Mean^a^	Change^b^	n^c^	*p-Value*^d^
K-12	13.9 ± 0.4	/	248	—	26.4 ± 0.9	/	278	—	12.7 ± 0.3	/	264	
moaD	15.1	8.7%	11	0.0076	31.3	18.5%	106	<0.0001	13.2	/	96	
JW09292	14.6	5.2%	131	<0.0001	28.6	8.2%	116	<0.0001	13.1	/	116	
fdoG	12.9	/	93		30.4	15.1%	107	<0.0001	13	/	91	
JW1330	14.5	/	124		28.5	7.9%	109	0.0315	12.6	/	73	
Lar	13.5	/	99		29.4	11.5%	109	<0.0001	12.6	/	125	
panE	14.3	/	96		30.6	15.9%	105	<0.0001	10.7	/	140	
mdoH	14.1	/	109		28.2	6.7%	113	0.029	11.8	/	146	
yjgB	13.1	/	131		28.2	11.3%	109	<0.0001	12.2	/	137	
elaA	12.7	/	83		30.1	14%	105	<0.0001	13.2	/	100	
kdpB	15.6	11.9%	140	0.0375	29.4	11.4%	106	<0.0001	12.6	/	99	
dcuC	13.6	/	94		28.7	8.8%	118	0.0013	12.7	/	112	
mutS	11.3	/	69		29.5	11.7%	104	<0.0001	12.2	/	94	
htrG	13	/	112		27.6	/	108		12.4	/	140	
dhaH	11.8	/	92		29.4	11.3%	111	<0.0001	12.5	/	99	
rimK	12.7	/	117		30.2	14.4%	108	<0.0001	13.2	/	127	
gloB	13.2	/	112		29.2	10.8%	109	<0.001	12.4	/	94	
clpX	15.9	14.4%	96	<0.0001	32.5	23%	109	<0.0001	12.6	/	79	
tufA	12.7	/	123		29.7	12.4%	107	<0.0001	12.6	/	87	
ybeA	13.4	/	126		28.1	6.6%	110	0.02	13.2	/	82	
JW0632	13.4	/	119		28.3	7.3%	108	0.018	13.9	/	129	
rfaD	14.8	6.2%	128	0.45	30.9	16.9%	106	<0.0001	13.2	/	127	
yjiK	13.1	/	123		30.8	16.8%	107	<0.0001	12.3	/	105	
fdoH	12.7	/	84		28.6	8.3%	110	<0.0001	12.1	/	103	
yncA	12.6	/	83		31.2	18.2%	106	<0.0001	12.4	/	89	
fepB	16.7	19.8%	89	<0.0001	32	21.3%	90	<0.0001	12.3	/	146	
hsdS	14.9	7.3%	112	0.0172	29.3	11%	108	<0.0001	13.4	/	128	
ygiQ	13.8	/	108		29.2	10.7%	108	<0.0001	12.8	/	56	
ung	12.2	/	77		29	9.7%	110	<0.0001	12	/	92	
hdfR	14.7	/	141		27.6	/	112		12.8	/	135	
yjbD	13	/	99		29.4	11.3%	112	<0.0001	13.8	8.20%	124	<0.0001
fepC	15.6	12.4%	150	<0.0001	31.5	19.3%	106	<0.0001	12.9	/	126	
hypE	14.4	/	135		26.8	/	98		12.7	/	145	
JW5726	15.3	10.4%	115	0.0125	26.9	/	103		12.3	/	102	
ydhF	14.3	/	205		26.8	/	107		12.2	/	113	
yqeH	15.1	8.3%	141	0.0266	25.6	/	98		12.4	/	139	
yjgN	14.3	/	101		26.4	/	113		12.2	/	150	
JW1321.5	13.4	/	135		25.7	/	106		12.3	/	142	
cyaA	18.9	36.3%	135	<0.001	23.4	/	53		13	/	144	
moaE	15.5	12.2%	116	0.0015	26	/	53		12.2	/	90	
sufA	14.7	/	129		28.1	6.5%	103	<0.0001	12.5	/	105	
sufE	15.2	9.4%	133	0.02	27.4	/	105		12.2	/	128	
yggX	15.2	9.3%	145	0.0137	26.3	/	107		12.6	/	146	
yheM	16.6	19.3%	141	<0.0001	28.3	7.3%	104	<0.0001	11.8	/	105	
yedR	15.2	9.5%	143	0.021	26.2	/	107		12.3	/	84	
fepG	14.4	/	83		28.8	9%	103	<0.0001	11.8	/	139	
yicM	15.9	14.2%	146	<0.0001	27.1	/	101		12.6	/	139	
dmsC	15.8	19.2%	129	<0.0001	23.3	8.8%	103	<0.0001	11.89	/	102	
yjgX	14.8	/	179		25.6	/	111		12.3	/	144	
yrfD	14.8	/	172		27.4	/	110		12.4	/	144	
srlA	14.9	/	131		22.2	/	72		11.6	/	121	
pepA	15.2	9.7%	137	0.0119	25.7	/	110		12.1	/	113	
yfaS	13.2	/	97		26.8	/	106		12.5	/	128	
yjhc	14.4	/	98		28.2	6.9%	108	0.0258	13.5	6.40%	127	0.0039
tolA	16.8	21.1%	162	<0.0001	27.7	5%	107	<0.0001	13.5	6.30%	122	<0.0001
ygjQ	15.1	8.7%	143	0.035	26.3	/	107		12.7	/	127	
yqjH	15.3	10%	113	0.0074	26.6	/	107		12.5	/	124	

Lifespan of *C. elegans* when feeding on bacterial knockouts: All experimental lifespan assays were performed in triplicate and survival data was grouped. Control lifespan assays performed at 2–3 different time points were done in triplicate and the survival curves were averaged and used to compare to experimental lifespan sets.

^a^The mean lifespan in days.

^b^The percent change of mean lifespan relative to K-12 control lifespan.

^c^The total number of individuals scored.

^d^The *p-*value of log-rank test comparing survival curves of gene knockout fed to control strain fed worm.
