# Supplementary material for: A genome-wide screen of bacterial mutants that enhance dauer formation in C. elegans
Source: Sci Rep. 2016 Dec 13;6:38764. doi: 10.1038/srep38764 (PMC5153853; doi:10.1038/srep38764)
Supplement: Supplementary Data [file srep38764-s1.doc]

**Supplementary Information:**

**A genome-wide screen of bacterial mutants that enhance dauer formation in *C. elegans***

Amit Khanna^1,#^, Jitendra Kumar^1,#^, Misha A. Vargas^1,#^, LaKisha Barrett^1^, Subhash Katewa^1^, Patrick Li^1^, Tom McCloskey^1^, Amit Sharma^1^, Nicole Naudé^1^, Christopher Nelson^1^, Rachel Brem^1^, David.W. Killilea^2^, Sean Mooney^3^, Matthew Gill^4^ and Pankaj Kapahi^1^*****

^1^ Buck Institute for Research on Aging, 8001 Redwood Blvd, Novato, USA

^2^ Nutrition & Metabolism Center, Children’s Hospital Oakland Research Institute, 5700 Martin Luther King Jr. Way, Oakland, CA, USA

^3^ Department of Biomedical Informatics and Medical Education, University of Washington, Seattle, Washington 98195, USA

^4^ Department of Metabolism & Aging, The Scripps Research Institute- Scripps Florida, Jupiter, Florida, 33458, USA

^#^These authors contributed equally

**Buck Institute for Research on Aging**

**8001 Redwood Blvd,**

**Novato, CA 94945**

**Tel: 415-209-2201**

***Corresponding author**

**E-mail Pkapahi@buckInstitute.org**

**Supplementary Figures**

**Figure S1.**

**
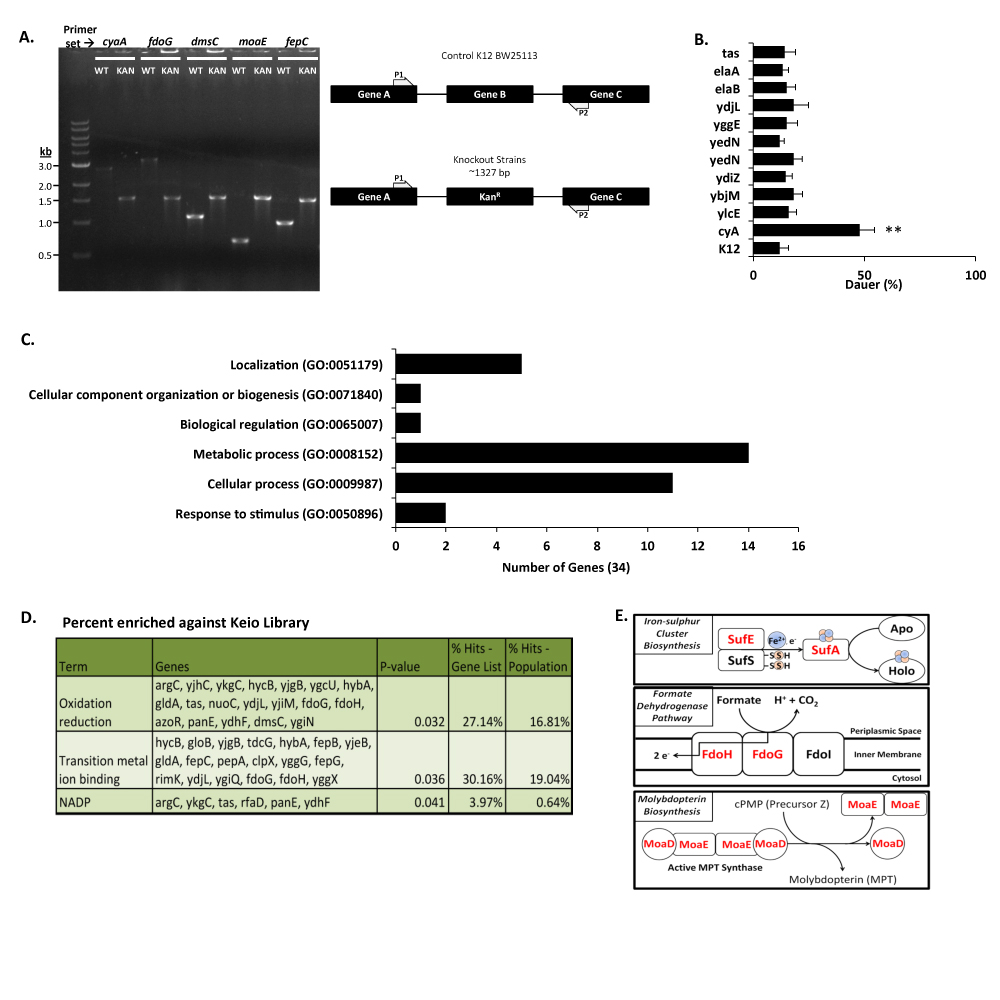
**

**Figure S1: Representative PCR for kanamycin cassette insertion and primer design. (A)** PCR products of the K-12 control and 5 representative knockout strains run on a 1% agarose gel and visualized with ethidium bromide. Kan cassette insertion into the genome was verified by growth in kan-LB media and by PCR with neighboring gene locus specific primers (P1, P2), as described in the text. EcoCyc confirmed K-12 wild-type gene size. **(B)** Dauer formation of *daf-2(e1370)* fed on K12 bacteria and mutant bacteria that were not identified from the primary screen as dauer enhancers. In each case, the data is represented as mean percent ± S.D of three replicates, **, *P*<0.0001, n>200. **(C)** The biological function analysis of candidate genes was done using PANTHER pathway database. **(D)** GO enrichment of bacterial mutants that enhance dauer formation from the screen using DAVID. **(E)** The *suf* pathways fall under transcriptional regulation. *fdo* and *moa* genes fall within transcriptional operons.

**Figure S2.**

**
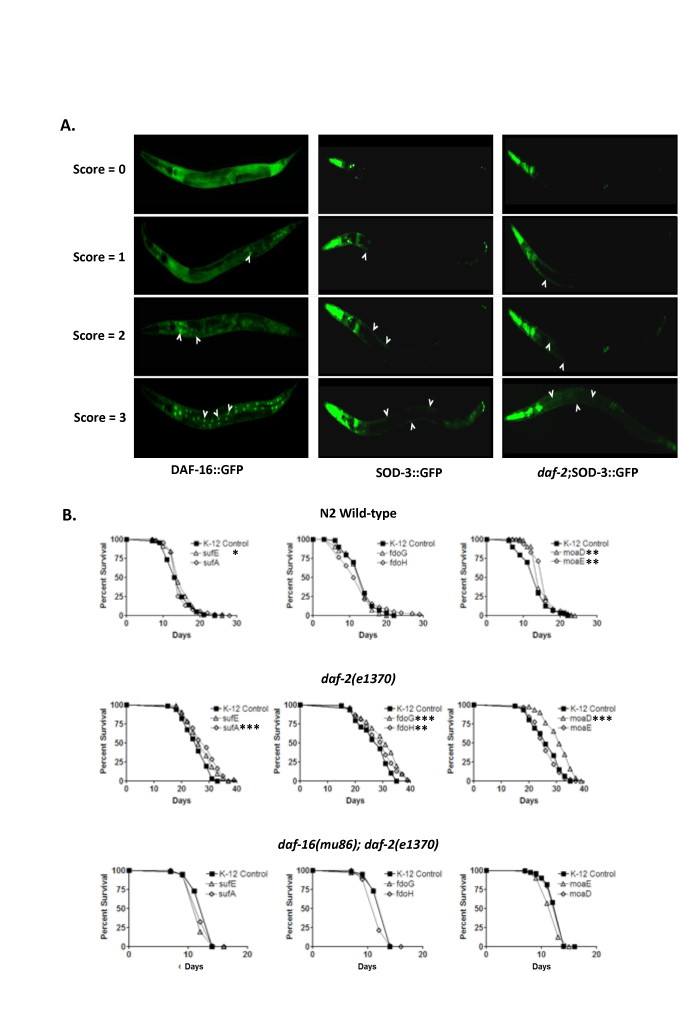
**

**Figure S2: Representative images for DAF-16 activation and lifespan phenotypes upon exposure to various bacterial mutants. (A)** Representative images corresponding to individual scores of DAF-16 activation. Scores relative to respective controls were given with increasing localization or expression of GFP (score 0-3) using the DAF-16::GFP, SOD-3::GFP, and DAF-2;SOD-3::GFP. **(B)** Representative lifespan curves of *C. elegans* fed on dauer enhancing bacterial mutants*.* Representative lifespan curves in 3 genetic backgrounds shown in Table 2. Significance determined by log-rank test using Prism 4 software.

**Figure S3**

**
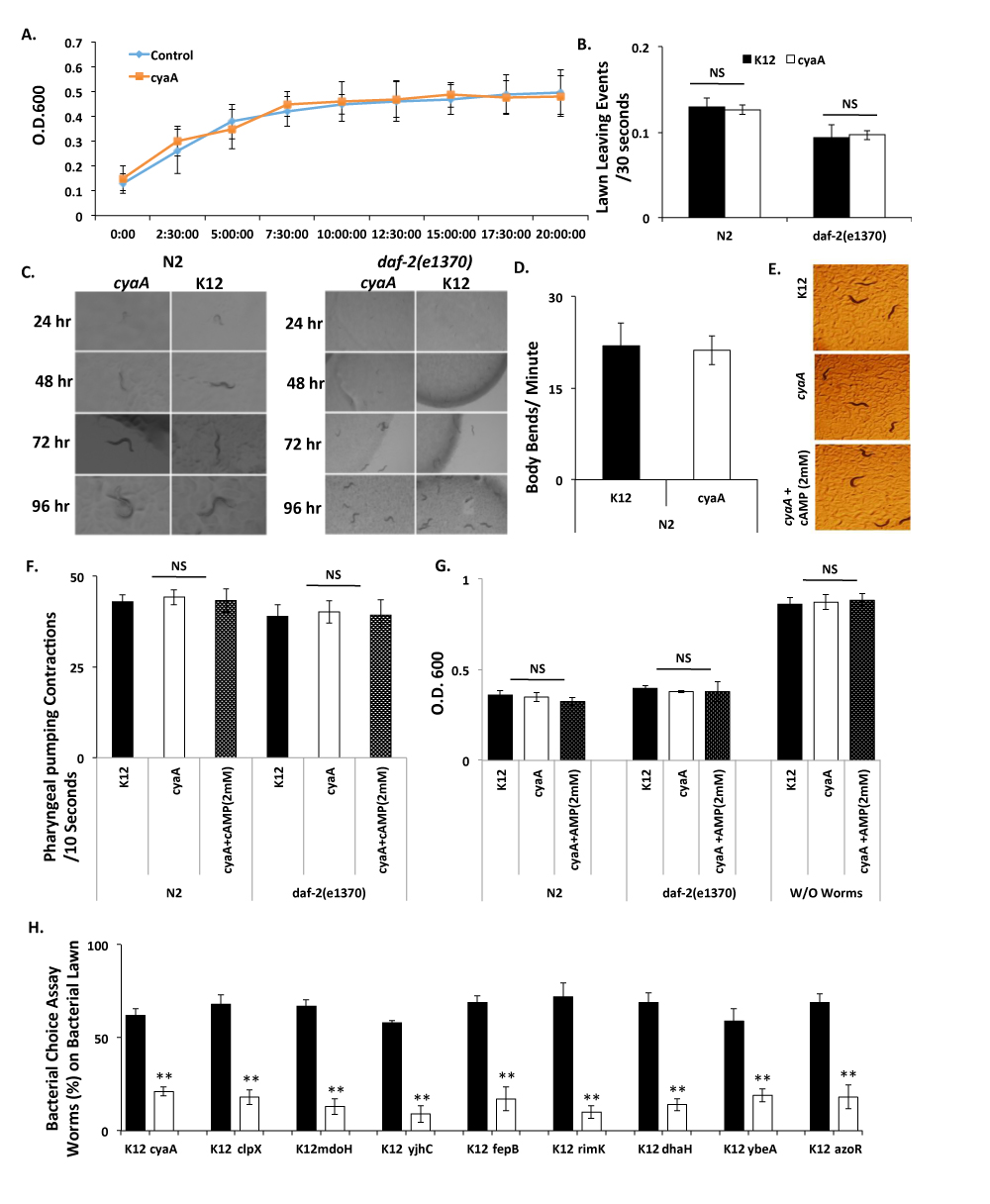
**

**Figure S3: Effect of *cyaA* bacterial mutant on worm physiology and behavior. (A)** No significant change was observed in bacterial growth curves of *cyaA* mutant bacteria as compared to K-12 **(B-G)** Worms fed on *cyaA* mutant bacteria or supplemented with exogenous cAMP did not show any significant change in lawn leaving behavior **(B)**, physiology **(C&E)**, body bends per minute **(D)**, pharyngeal pumping **(F)**, and feeding behavior based on the bacterial depletion after overnight feeding **(G)**. (**H**) Bacterial choice assay was performed between 9 dauer enhancing bacteria from Table 1 and K12 control strain. Histogram showing the percentage of worms on bacterial lawn indicated. The data is represented as mean percent ± S.D of three replicates. n > 200 **, *P*<0.01

**Figure S4**

**
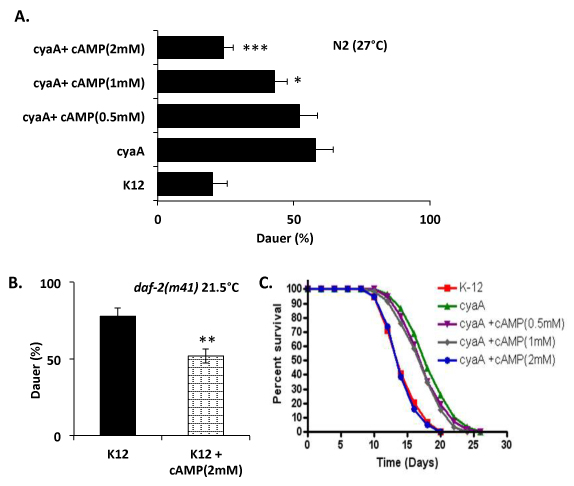
**

**Figure S4: Exogenous cAMP rescues dauer formation and lifespan in a dose dependent manner. (A)** A reduction in dauer formation (N2) was observed in a dose dependent manner when bacterial lawns were supplemented with cAMP. **(B)** A reduction in dauer formation in *daf-2(m41)* mutants was observed upon addition of 2mM cAMP to the K12 bacterial lawn. **(C)** A dose dependent change in lifespan was observed in N2 strain when *cyaA* bacterial lawns were supplemented with cAMP. n > 200, **P*<0.005, ** *P*<0.01, ****P*<0.0001

**Figure S5**

**
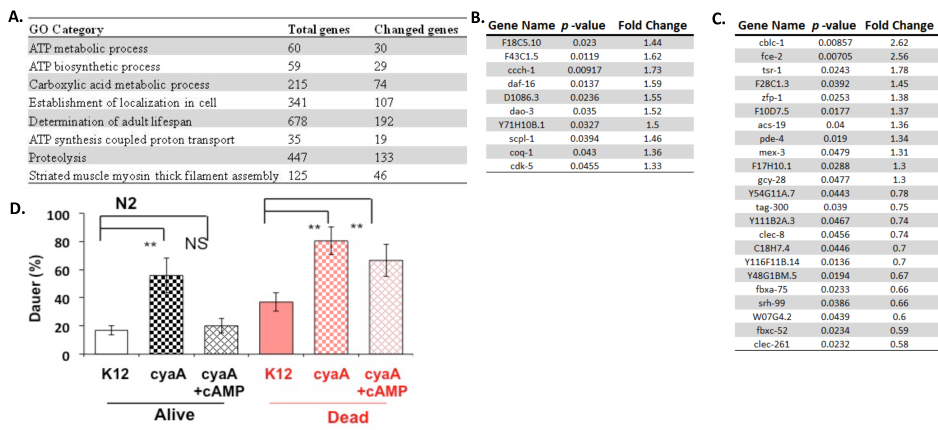
**

**Figure S5: *C. elegans* response upon feeding *cyaA* bacteria compared to K-12. (A)** Functional classification of genes with significant differential expression in worms fed on *cyaA* compared to K-12 bacteria using microarray analysis. The biological functions and interactions of genes, the candidate genes were analyzed using KEGG pathway database. **(B-C)** Representative list of DAF-16 dependent (**B**) and independent (**C**) genes summarized as a fold change difference. **(D)** The effect of live (left) *vs* dead (right) *cyaA* bacterial mutant on *C. elegans* dauer formation. Both dead and alive bacterial mutants of *cyaA* enhance dauer formation in control animals (N2). However, supplementing with 2mM cAMP only suppresses dauer formation in worms fed on live bacteria (left). Dauer assay was performed at 27°C. Data is represented as mean percent ±SD of greater than 3 biological replicates, n > 200. ** *P*<0.01

| **Table S1 Statistical analysis of dauer assay experiments** | | | | |  |  |
| --- | --- | --- | --- | --- | --- | --- |
|  |  | |  |  |  |  |
| **Genotype** | **Bacteria ^a^** | | **Chemical** | **Dauer** | **n ^b^** | ***p* ^c^** |
|  |  |  |  | **Average** |  |  |
|  | | | | | | |
| N2 | | K12 | / | 7.02 | 205 | 0.00 |
| N2 | | cyaA | / | 55.21 | 224 |  |
| N2 | | K12 | cAMP | 6.61 | 201 | 0.03 |
| N2 | | cyaA | cAMP | 23.17 | 239 |  |
| *daf-11(m47)* | | K12 | / | 8.02 | 235 | 0.04 |
| *daf-11(m47)* | | cyaA | / | 10.32 | 214 |  |
| *daf-11(m47)* | | K12 | cAMP | 7.61 | 222 | 0.03 |
| *daf-11(m47)* | | cyaA | cAMP | 11.17 | 227 |  |
| *daf-2(e1370)* | | K12 | / | 13.20 | 236 | 0.00 |
| *daf-2(e1370)* | | cyaA | / | 49.43 | 239 |  |
| *daf-2(e1370)* | | K12 | cAMP | 12.45 | 203 | 0.03 |
| *daf-2(e1370)* | | cyaA | cAMP | 23.17 | 245 |  |
| *daf-7(e1372)* | | K12 | / | 48.20 | 245 | 0.05 |
| *daf-7(e1372)* | | cyaA | / | 50.43 | 239 |  |
| *daf-7(e1372)* | | K12 | cAMP | 49.45 | 238 | 0.01 |
| *daf-7(e1372)* | | cyaA | cAMP | 43.17 | 241 |  |
| *daf-2(e1370);daf-3(e1376)* | | K12 | / | 59.20 | 231 | 0.04 |
| *daf-2(e1370);daf-3(e1376)* | | cyaA | / | 62.43 | 201 |  |
| *daf-2(e1370);daf-3(e1376)* | | K12 | cAMP | 58.45 | 219 | 0.23 |
| *daf-2(e1370);daf-3(e1376)* | | cyaA | cAMP | 53.17 | 234 |  |
| *daf-2(e1370);daf-5(e1386)* | | K12 | / | 10.20 | 224 | 0.32 |
| *daf-2(e1370);daf-5(e1386)* | | cyaA | / | 13.43 | 228 |  |
| *daf-2(e1370);daf-5(e1386)* | | K12 | cAMP | 13.45 | 217 | 0.03 |
| *daf-2(e1370);daf-5(e1386)* | | cyaA | cAMP | 11.17 | 208 |  |

**Dauer Assays were performed at conditions as indicated in the results section**.

^a^ The mean percentage of dauer staged animals.

^b^ The total number of individuals scored (includes all replicates)

^C^ The *p*-value for a student’s two-tailed t-test comparing cyaA/+/-cAMPknockout strain to control strain.

| **Table S2 Statistical analysis of lifespan experiments** | | | | |  |  |  |
| --- | --- | --- | --- | --- | --- | --- | --- |
|  |  |  |  |  |  |  |  |
| **Genotype** | **Bacteria ^a^** | **Chemical** | **Lifespan (days)** | | **Percent Change ^b^** | **n ^c^** | ***p* ^d^** |
|  |  |  | **Mean** | **Max** |  |  |  |
| N2 | K12 | / | 14.19 | 21 | / | 178 | / |
| N2 | cyaA | / | 16.42 | 25 | 19.05 | 162 | <0.0001 |
| N2 | K12 | cAMP | 12.42 | 19 | -9.52 | 189 | <0.0001 |
| N2 | cyaA | cAMP | 14.11 | 20 | -4.76 | 156 | <0.0001 |
| *daf-11(m47)* | K12 | / | 27.91 | 34 | / | 189 | / |
| *daf-11(m47)* | cyaA | / | 27.88 | 34 | 0.00 | 178 | 0.21 |
| *daf-11(m47)* | K12 | cAMP | 26.09 | 34 | 0.00 | 187 | 0.21 |
| *daf-11(m47)* | cyaA | cAMP | 27.76 | 34 | 0.00 | 173 | 0.41 |
| *daf-2(e1370)* | K12 | / | 28.51 | 34 | / | 182 | / |
| *daf-2(e1370)* | cyaA | / | 25.45 | 37 | 8.82 | 190 | <0.0001 |
| *daf-2(e1370)* | K12 | cAMP | 21.39 | 30 | -11.76 | 178 | <0.0001 |
| *daf-2(e1370)* | cyaA | cAMP | 23.22 | 31 | -8.82 | 189 | <0.0001 |
| *daf-7(e1372)* | K12 | / | 21.70 | 29 | / | 178 | / |
| *daf-7(e1372)* | cyaA | / | 21.59 | 28 | -3.45 | 154 | 0.23 |
| *daf-7(e1372)* | K12 | cAMP | 21.17 | 29 | 0.00 | 164 | 0.34 |
| *daf-7(e1372)* | cyaA | cAMP | 21.02 | 28 | -3.45 | 178 | 0.31 |
| *daf-2(e1370);daf-3(e1376)* | K12 | / | 26.91 | 35 | / | 190 | / |
| *daf-2(e1370);daf-3(e1376)* | cyaA | / | 26.18 | 35 | 0.00 | 184 | 0.34 |
| *daf-2(e1370);daf-3(e1376)* | K12 | cAMP | 25.23 | 34 | -2.86 | 183 | 0.43 |
| *daf-2(e1370);daf-3(e1376)* | cyaA | cAMP | 26.56 | 35 | 0.00 | 181 | 0.32 |
| *daf-2(e1370);daf-5(e1386)* | K12 | / | 21.45 | 33 | / | 182 | / |
| *daf-2(e1370);daf-5(e1386)* | cyaA | / | 23.58 | 32 | -3.03 | 187 | 0.75 |
| *daf-2(e1370);daf-5(e1386)* | K12 | cAMP | 23.29 | 33 | 0.00 | 167 | 0.76 |
| *daf-2(e1370);daf-5(e1386)* | cyaA | cAMP | 22.76 | 32 | -3.03 | 178 | 0.53 |
| *daf-16(mu86)* | K12 | / | 11.70 | 16 | / | 178 | / |
| *daf-16(mu86)* | cyaA | / | 11.59 | 16 | 0.00 | 173 | 0.42 |
| *daf-16(mu86)* | K12 | cAMP | 11.17 | 16 | 0.00 | 153 | 0.42 |
| *daf-16(mu86)* | cyaA | cAMP | 11.02 | 16 | 0.00 | 142 | 0.32 |

**Lifespan of *C. elegans* when feeding on bacterial knockouts:** All experimental lifespan assays were performed in triplicate and survival data was grouped. Control lifespan assays performed at 2-3 different time points were done in triplicate and the survival curves were averaged and used to compare to experimental lifespan sets.

^a^ The mean lifespan in days.

^b^ The percent extension of mean lifespan relative to K-12 control lifespan

^c^ The total number of individuals scored.

^d^ The *p-*value of log rank test comparing survival curves of gene knockout fed to control strain fed worms
